# Implementation Science and Employer Disability Practices: Embedding Implementation Factors in Research Designs

**DOI:** 10.1007/s10926-016-9677-7

**Published:** 2016-10-28

**Authors:** Chris J. Main, Michael K. Nicholas, William S. Shaw, Lois E. Tetrick, Mark G. Ehrhart, Glenn Pransky, the Hopkinton Conference Working Group on Workplace Disability Prevention, Benjamin C. Amick, Johannes R. Anema, Elyssa Besen, Peter Blanck, Cécile R. L. Boot, Ute Bültmann, Chetwyn C. H. Chan, George L. Delclos, Kerstin Ekberg, Mark G. Ehrhart, Jean-Baptiste Fassier, Michael Feuerstein, David Gimeno, Vicki L. Kristman, Steven J. Linton, Chris J. Main, Fehmidah Munir, Michael K. Nicholas, Glenn Pransky, William S. Shaw, Michael J. Sullivan, Lois E. Tetrick, Torill H. Tveito, Eira Viikari-Juntura, Kelly Williams-Whitt, Amanda E. Young

**Affiliations:** 1 Arthritis Care UK Primary Care Center, Keele University, North Staffordshire, UK; 2 Pain Management Research Institute, Sydney Medical School – Northern, Royal North Shore Hospital, St. Leonards, NSW 2065 Australia; 3 Liberty Mutual Research Institute for Safety, Hopkinton, MA USA; 4 University of Massachusetts Medical School, Worcester, MA USA; 5 George Mason University, Fairfax, VA USA; 6 San Diego State University, San Diego, CA USA

**Keywords:** Implementation factors, Workplace interventions, Disability prevention, Research priorities

## Abstract

*Purpose* For work disability research to have an impact on employer policies and practices it is important for such research to acknowledge and incorporate relevant aspects of the workplace. The goal of this article is to summarize recent theoretical and methodological advances in the field of Implementation Science, relate these to research of employer disability management practices, and recommend future research priorities. *Methods* The authors participated in a year-long collaboration culminating in an invited 3-day conference, “Improving Research of Employer Practices to Prevent Disability”, held October 14–16, 2015, in Hopkinton, MA, USA. The collaboration included a topical review of the literature, group conference calls to identify key areas and challenges, drafting of initial documents, review of industry publications, and a conference presentation that included feedback from peer researchers and a question/answer session with a special panel of knowledge experts with direct employer experience. *Results* A 4-phase implementation model including both outer and inner contexts was adopted as the most appropriate conceptual framework, and aligned well with the set of process evaluation factors described in both the work disability prevention literature and the grey literature. Innovative interventions involving disability risk screening and psychologically-based interventions have been slow to gain traction among employers and insurers. Research recommendations to address this are : (1) to assess organizational culture and readiness for change in addition to individual factors; (2) to conduct process evaluations alongside controlled trials; (3) to analyze decision-making factors among stakeholders; and (4 ) to solicit input from employers and insurers during early phases of study design. *Conclusions* Future research interventions involving workplace support and involvement to prevent disability may be more feasible for implementation if organizational decision-making factors are imbedded in research designs and interventions are developed to take account of these influences.

Work disability is a key health outcome measure that is of critical lifestyle importance to workers who suffer pain, impairment, and chronic illness [1]. In addition to the negative health implications of being out of work [2], the cost of supporting disabled workers has been rapidly growing in much of the industrialized world [3]. This trend underscores the importance of continued research into the individual, organizational, societal, and health care factors that affect an individual’s ability to find employment, stay at work, or return to work after the onset of health problems. Of particular importance are the workplace conditions, job demands, social and organizational support, and job accommodation and flexibility offered by employers [4]. Despite research evidence that workplace efforts are critical for preventing disability, promoting the implementation of new disability management policies and procedures within organizations has posed many challenges [5–7].

With the goal of improving future research of employer disability prevention strategies, the authors participated in an invited 3-day conference, “Improving Research of Employer Practices to Prevent Disability”, held October 14–16, 2015, in Hopkinton, Massachusetts, USA. Methods and general proceedings of the conference are described in the introductory article to this special issue [4]. The authors of the present article represented a sub-group tasked with understanding current trends in Implementation Science and its relevance with respect to employer practices for managing and preventing disability. We were asked to review the applicable scientific literature, assess its relevance for employer decision-making, compare implementation factors described in the scientific and employer-directed “grey literature”, contrast key conceptual and theoretical frameworks, and recommend future research priorities.

## Workplace-Focused Interventions: the Case of Musculoskeletal Disorders

The management of musculoskeletal disorders provides a good example for exploring implementation issues; it is an area in which the issue of work disability has been a particular concern and where researchers have concluded early patient-centered and workplace-focused approaches are needed to improve return-to-work (RTW) outcomes [8, 9]. Guidelines for return to work following light to moderate (soft tissue or musculoskeletal) workplace injuries consistently recommend the need for early diagnostic triage, identification of potential psychosocial obstacles to recovery, provision of advice that these are self-limiting conditions and, importantly, that remaining at work or an early RTW with temporary job modifications should be encouraged and supported [10–13]. Despite evidentiary support for these practices, there is considerable variation in application and outcomes [14, 15]. Possible explanations for these variations have come from a range of sources. A review of controlled trials [16], for example, revealed that when psychological obstacles to recovery (so-called ‘yellow flags’) are identified and treatment is directed at their amelioration, better disability and RTW outcomes can be achieved than by providing the same approach to all injured workers. There is further evidence that when health care providers follow the recommended guidelines and have direct contact with injured workers’ employers, they achieve better RTW outcomes [17, 18].

Involvement of the workplace is of crucial importance. For example, Linton [19] and Shaw [15], found that teaching supervisors basic communication skills (e.g. negotiating accommodations) had promising benefits for workers with persisting pain problems. Supervisor training in communication and problem-solving skills (for both injured workers with persisting back pain and their supervisors) has been shown to achieve significant benefits in terms of reduced work absence due to pain, perceived health, and reduced health-care utilization [20]. Despite research support, engaging the workplace as part of the treatment or intervention process is still the exception rather than the rule [21], and positive workplace support and job modification are not easily achieved. Thus, in a controlled trial of a guidelines-based intervention (i.e., early contact with absentees, addressing psychosocial obstacles, modified work offers, communication among stakeholders), implementation of the experimental intervention was impeded by unforeseen organizational obstacles (failure to implement the absence management protocol at one experimental site), and this had detrimental effects on measured outcomes across groups [22]. Clearly, it cannot be assumed that the workplace is always a neutral or benign environment as far as implementation of RTW processes is concerned.

Previous analyses of the role of the workplace in enhancing RTW outcomes have identified a range of contributing factors, such as the perspective held of the workplace by the injured workers [23], features of the workplace and its responsiveness to the injured worker [24], and the need to accommodate the differing interests held by the range of stakeholders who are involved [25] (see accompanying articles on workplace factors and interventions [26, 27]). Achieving a successful RTW is likely therefore to require workplace changes at several levels. Accordingly, we need to consider what these might be and how they might be achieved.

## Contributions of Implementation Science (Imp Sci)

Imp Sci is a new and growing field of research focusing on the methods that influence integration of evidence-based interventions into practice settings [28]. Though much of the early research in this field has focused on implementation of innovations within healthcare and education, the essential principles and conceptual frameworks may be relevant to understanding adoption of evidence-based work disability prevention efforts among employers and insurers. However the field includes the study of organizational behavior and in terms of conceptualisation, methodology and measurement contains much of direct relevance to the management of work disability in particular.

Implementation can be seen as part of a continuum from *diffusion* (the passive, untargeted and un-planned spread of new practices), to *dissemination* (the active spread of new practices to the target audience using planned strategies), and finally, *implementation* (the process of putting to use or integrating new practices within a setting) [29, 30]. There are at least three overarching aims in the use of theories, models and frameworks in Imp Sci: (1) describing and/or guiding the process of translating research into practice, (2) understanding and/or explaining what influences implementation outcomes and (3) evaluating implementation [31]. Two key elements in implementation at the workplace are managerial decision-making and knowledge translation.

### Managerial Decision-Making

Implementation is inextricably linked with management. Organizational science has adopted evidence-based practice principles, and as a result, Imp Sci has tended to take what has been described as a predominantly ‘rational’ (or structured problem-solving) approach. Thus the decision maker should first identify the problem, then search for and generate alternative courses of action, implement the option selected, and then evaluate the outcome [32]. It has been assumed that acknowledgement of evidence in the ‘science-informed practice of management’ [33], is not only desirable and perhaps necessary, but also sufficient. However, numerous authors have argued that this view requires qualification. For example, Baba and Hakem Zadeh [34] described a model in which evidence based on judgment, education and experience affects the decision-makers’ options as well as their actual decision, and in which such decisions are moderated by context, management preferences and values, as well as by stakeholders’ preferences and values. It is important also to consider the decision process, characteristics of the decision-maker and the context when implementing evidence-based management practices [35]. It has also been shown that decision makers may deviate from a strictly ‘rational’ approach in weighting the current status more strongly or seek the minimum requirements needed to satisfy choices rather than necessarily optimizing outcomes [36]. Further, in Wright et al.’s analysis [35], decisions are often made in a social environment allowing for political bargaining [37] and other communications between participants [38]. Based on their study of a program implemented in an emergency department in Australia, Wright et al. [35] found that the implementation process began with problem recognition and then proceeded to assembling evidence before exchanging evidence across disciplines and decision-makers. This resulted in reformulating the problem, engaging stakeholders and generating alternatives. This process resulted in commitment to the evidence-based solution and implementation. Wright et al. [35] conclude that it is important to recognize “situated expertise” among decision-makers and people who are actually implementing a new program or policy. (By situated expertise they are referring to the proficiency and judgment that individuals have as a result of their experiences, education and practice.)

Viewing implementation as a decision process emphasizes that the decision-making process and actions occur in the context of the involvement of multiple individuals within the organizations, and possibly, stakeholders outside of the organization. The widespread use of teams within organizations suggests that the project management literature may be informative in understanding the process of implementation, especially in the initial stages, although, project teams tend to have a specific goal and a limited life as opposed to the apparently unlimited time line of regular organizational staff [39]. In order to improve the implementation processes, implementation teams need to seek feedback, experiment, and discuss errors that are made [40]. Finally, Horwitz [41] proposes that functional diversity in project teams, and we would argue, in implementation teams, improves the likelihood of successful implementation due to likely greater flexibility. In the next section we will consider the contribution of knowledge translation.

### Knowledge Translation

The advancement of the science of knowledge translation or how to most effectively promote and support the use of evidence in health and healthcare policy and practice, is challenged by the plethora of terms, models, frameworks, and heterogeneous interventions employed in the field [42]. Broadly, knowledge translation is the synthesis, dissemination, exchange and ethically sound application of knowledge to improve health and well-being [43]. It offers a “technology” for change which is potentially applicable to the management of work disability, but it is necessary to ask how this might be achieved.

There have been many attempts to systematise interventions. The Cochrane Effective Practice and Organization of Care (EPOC) Group [44], for example, is a widely used classification scheme, but recently, in a scoping review of interventions, Lokker et al. [42] identified 51 diverse classification schemes, and described them in terms of content, focus and methodology. The *content* areas include dissemination and implementation, knowledge translation, quality improvement, knowledge transfer and research utilization. Lokker et al. [42] updated an earlier review [45] by including policy articles and adding search terms related to knowledge translation to capture broader classification schemes. There have been attempts to simplify the field of Imp Sci by developing some broad, over-arching frameworks. One of the better known is the Consolidated Framework for Implementation Research (CFIR) [46].

## The Consolidated Framework for Implementation Research (CFIR)

The CFIR offers a helpful framework for consolidating the influence of complex, interacting and multi-level factors, thus enabling a wide range of contextual factors to be considered by unifying key constructs from published implementation theories. We have adopted it for our analysis of employer disability prevention strategies.

The CFIR comprises five domains: intervention characteristics, outer setting, inner setting, characteristics of the individuals involved, and the actual process of implementation [46]. Further, there are a number of constructs related to the intervention (e.g. evidence strength): an outer setting (e.g. patient needs and resources), an inner setting (e.g. culture and leadership engagement), as well as individual and process variables (e.g. plan, evaluate, and reflect).

Building on this initial work, Aarons et al. [47] proposed a multi-level, phased model of the implementation process that derived from published studies. The model comprises four phases: Exploration, Adoption/Preparation, Implementation, and Sustainment (or EPIS). The multi-levels reflected the outer and inner contexts identified in the earlier Damschroder paper [46]. Aarons et al. [47] emphasized that their EPIS model should be seen as a framework that could describe variables hypothesized to play important roles in achieving effective implementation of evidence-based practice guidelines. In their case, Aarons et al. [47] were concerned with implementation issues in child welfare settings, but they indicated the model was not intended to apply only in that context. However, they did consider that it might be best suited to innovations within human service organizations rather than business or agriculture settings. Nevertheless, if we treat it at the conceptual level it would seem reasonable to examine its potential for a wider range of applications.

**Fig. 1:**
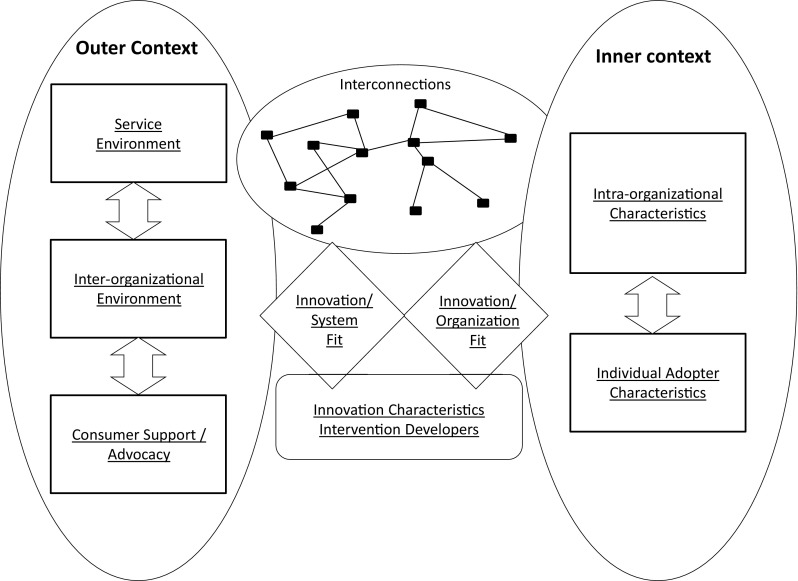
Key features of the CFIR model [45] (reprinted with permission)

The key features of the model are graphically portrayed in Fig. 1. By *outer* contexts, Aarons et al. [47] identify the social and political environment in which the organization (e.g. legislative and legal frameworks, as well as funding and networks between organizations). *Inner* contexts refer to the particular characteristics of the organization in question (e.g. leadership, culture, values and goals, as well as the characteristics of individuals within the organization). It is suggested that different aspects of these contexts might be more or less prominent at different phases of an implementation process, and that these might, in turn, influence succeeding aspects.

### Four Phases of Implementation

**Fig. 2:**
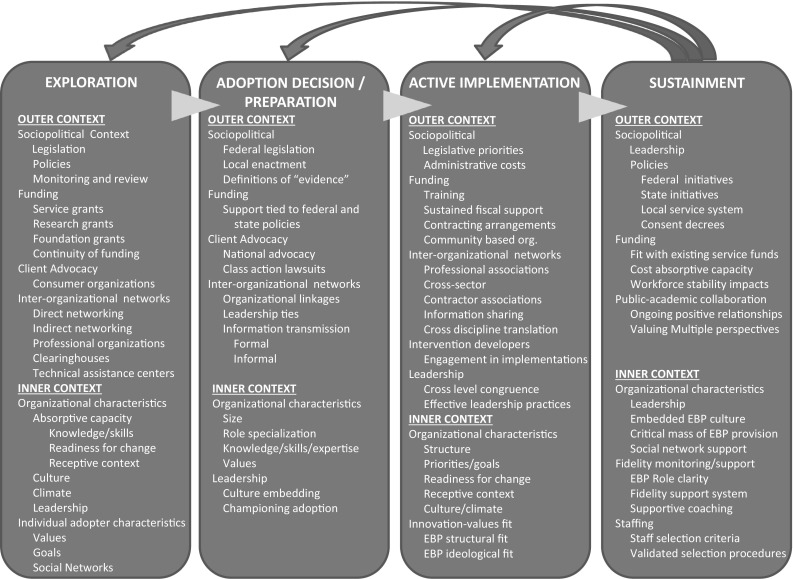
Detailed description of CFIR model components [45] (reprinted with permission)

The phases of implementation (EPIS) in the CFIR and possible contributions aligned with inner and outer contexts, are illustrated in Fig. 2. *Phase 1*, described as Exploration, is characterized by developing an awareness of an issue requiring attention (e.g. a desire for an improved approach to a problem) and should include consideration of the question in terms of the possible inner and outer contexts.

*Phase 2* in this model (Adoption/Preparation) refers to a literature search comprising a review of evidence for previous attempts to address this type of problem, as well as available resources that might be relevant to the current task. The main outcome of this phase should be to enable a decision to adopt the proposed innovations or changes that fits inner and outer contexts as well as possible thereby leading to agreement on a plan for implementation.

*Phase 3* comprises the Active Implementation of the plan, which is expected to require engagement at inner and outer contexts, as well as fostering supporting linkages between them. Aarons et al. [47] suggest that the scale of the implementation project is also likely to have important implications for specific issues in both outer and inner contexts. For example, at the outer level there may be large system issues, like funding availability, while at the inner level there may be issues that concern the fit with the productivity and other work demands of individual workers. Other inner context issues likely to require addressing might include: readiness to change (which may vary within an organization), the receptivity of the organizational culture to change, the current ‘Organizational climate’ (e.g. employees’ perceptions of their work environment), and how well the implementation plan fits with the existing values of the organization and its workers.

*Phase 4* is concerned with Sustainment of the intervention or the continued use of the intervention (or innovation) as standard practice within an organization. Aarons et al. [47] acknowledge that this aspect of their model has the least systematic supporting knowledge, with little empirical work on which to draw. Nevertheless, if the implementation is to move beyond mere demonstration, it is essential to consider sustainment. The problem of maintenance of change has long been recognized as a challenge in the clinical literature [48] and is reflected in the organizational sphere in the recognition that sustained return to work after injury may be of more relevance than speed of return to work (further discussion of outcome measures is offered in the companion paper [1]) and this may involve a series of changes as in the Organizational Readiness for Change (ORC) model [49], and the “self-regulation” model [50]. However, further empirical work on the determinants of change in the context of sustainment would seem to be merited.

### Utility of the CFIR Model

It often appears there is an assumption within intervention research that somehow the demonstration of an effect will be enough for others to take it up. In Imp Sci, researchers and the workplace must consider *how* the intervention can be maintained, with specification of these requirements and a major focus on actual implementation. This should make it more likely to achieve the important return on investment (ROI). Another key difference with much of the intervention literature for injured workers is that the CFIR model explicitly incorporates consideration of more than the presenting problem (e.g. back pain). It provides a framework for a range of distal and proximal workplace factors that may influence the outcome of a particular intervention and it acknowledges that these could have varying inputs at different stages of the RTW process.

To test the application of the Aarons et al. [47] classification model to disability prevention practices, we conducted a brief keyword search [“disability” and “accommodation” and “implementation”; “return to work” and “implementation”; (“presenteeism^1^” or “stay at work”) and “implementation”] that identified nine articles [25, 53–60], describing implementation factors related to absence management^2^ and RTW programs (one systematic review, four process evaluations, and four conceptual/theoretical summaries). Collectively, these 9 articles made mention of 89 factors influencing implementation, and we found these factors could be organized within the four-phased EPIS conceptual framework [47] without difficulty (Table 1). Examination of these factors shows similarity in the implementation issues raised by various researchers, and this sorting does suggest actionable problems that could be addressed.

**Table 1:** A sampling of implementation factors described in the return-to-work literature and fit to the CFIR conceptual framework

Phases			
Exploration	Adoption/preparation	Active implementation	Sustainment
*Inner context*			
Sr. management commitment Company size and sector People-oriented culture Safety-oriented culture Organizational bureaucracy Social bond with employees Responsibility for employees DM management priority Perceived effects on profitability Desire for cost containment Organizational readiness Pressure/need for change Concordant values Departmental linkages Good rapport with labor unions	Financial resources Experienced line managers Workforce awareness Nature of work tasks Pay grade systems Employees have meaningful voice Non-traditional work arrangements Prior experience with RTW issues Authority of case managers Institutional resources Staff perceptions of efficacy Organizational climate Training/coaching activities Biopsychosocial acceptance Conflicting internal practices	Feasibility of job modification Wage structure Departmental autonomy Budgetary arrangements External benefits Prompt identification Peer representatives Workers feel included in process Case manager training Sufficient resources Simple tools and processes Relationships with supervisors Mental demands of work Mental capacity of workers Timing of the intervention Accountability Administrative support Clarity of information Support for complex cases	Failure to follow guidelines Organizational awareness Departmental inconsistency Monitoring and review Access to specialist guidance Conflicts for line managers Valued career attachments Empowered supervisors Engaged case managers Level of complexity Feedback of program success Line managers deferring tasks Increased burden to co-workers Commitment of line managers
*Outer context*			
Legislative requirements Anticipated consequences Financial incentives Supportive legal frameworks Local economic trends Level of regulatory compliance Connection to profitability Diversity of stakeholder paradigms Communication with stakeholders Legal barriers to rehabilitation Perceived legitimacy of laws Legal barriers to collaboration Anti-discrimination legislation Legislated requirements for RTW Global financial downturn	Available rehabilitation services Existing occupational health service/provider that is well accepted by employees Internal arrangements to provide employees ready access to medical treatment and advice Availability of specialist advice Geographical proximity of HC providers Professional confidentiality as a barrier to shared information Legal or financial measures that reduce barriers to accessibility and accommodation	Problems getting timely medical care and evaluation Employer access to external guidance and support Employer access to specialist rehabilitative expertise Low level of physician input in RTW planning	Legislative support and incentives

## Incorporating Implementation Concepts when Developing a New Intervention

According to Graham et al. [61], “there is confusion and misunderstanding about the concepts of knowledge translation, knowledge transfer, knowledge exchange, research utilization, implementation, diffusion, and dissemination”, (p. 13). This diversity and inconsistency in terminology is a potential barrier to synthesizing, advancing, and applying the findings from what has been described as *knowledge translation* (KT) [62, 63].

It has been recommended that the basic unit of knowledge translation should usually be up-to-date systematic reviews or other syntheses of research findings and further that “Knowledge translators need to identify the key messages for different target audiences and to *fashion these in language and knowledge translation products that are easily assimilated by different audiences*” [64] (italics added for emphasis). They note further that the relative importance of knowledge translation to different target audiences will vary by the type of research and appropriate endpoints of knowledge translation may vary across different stakeholder groups [62, 63]. Lavis et al. [65] found that the key factors important to policy makers’ use of research evidence were: interactions between researchers and policy-makers (whether formal or informal); and the match of the research to the beliefs, values, interests, or political goals and strategies of elected officials, social interest groups, and others. (Abstracted in Grimshaw et al. [64]).

### Implementation Fidelity and Quality Improvement

Lack of fidelity during initial implementation may lead to underestimation of efficacy of the intervention and weaken the strength of conclusions [66]. Assessment of fidelity to behavioural interventions that require direct human observation and judgment has proved a challenge [67, 68]. However, technical solutions have been recommended to scale-up the evaluation and quantification of such behavioural interventions. For example, Atkins et al. [69] and Balsubramanian et al. [70] propose blending quality improvement and Implementation Research. This approach has been termed *Learning Evaluation,* in which qualitative and quantitative data are collected to conduct real-time assessment of implementation processes while also assessing changes in context, facilitating quality improvement using run charts, audit and feedback, and generating transportable lessons. If these principles could be applied across organizations they would merit consideration for adaptation to organisational settings.

## Implementation in the Workplace

There are not only many types of implementation but also a wide range of context-specific influences. In tackling work disability specifically, there are challenges not only at the level of individual workers, but also in the nature of the organization in which they work and in the interaction between these two spheres.

### Organizational Culture and Climate

*Organizational culture* has been defined as the *shared values, assumptions, and beliefs* that are communicated in the behaviors that the organization uses to overcome prior problems, thereby validating the importance of these actions [71, 72]. More specifically the distinction has been made between *artefacts* (or the most visible or easily accessed layer, such as people’s dress or the physical environment; the meaning or significance of which may vary from organization to organization); the *espoused values* (which may or may not be consistent with how the organization actually operates) and finally, the deepest layer comprising the *underlying assumptions* that are typically shared throughout the organization and that drive how employees interact and behave, Thus the concept of organizational culture is generally quite broad, encompassing all of these layers and almost all aspects of organizational life. Research has suggested the importance of a number of dimensions of organizational culture for implementation success across a variety of settings [73–78].

*Organizational climate* has been defined as the shared perception of the work environment including the policies, practices, and procedures that guide the expected, supported, and rewarded behaviours [71, 79, 80]. Although some climate researchers examine the general work environment that employees experience (or the molar climate [81]), when specific strategic outcomes are of interest), it may be helpful to adopt a more specific focus [80, 82]). In recent years, there has been a growing interest in the concept of implementation climate. *Implementation climate* [83, 84] is a global construct consisting of items related to expectations, support and rewards and has been suggested as an integrative framework linking the organisation and the worker [46]. It has been defined as “employees’ shared perceptions of the importance of innovation implementation within the organization” [84] (p. 813) and captures the expectations, support, and rewards associated with implementation [85]. Multiple measures of implementation climate have been developed [81, 84, 85], with generally supportive evidence for their reliability and validity.

In summary, implementation climate involves employee perceptions of *what* happens in the organization and implementation culture focuses on *why* it happens [86]. Unfortunately, while these may have explanatory utility, to date, outcomes of attempts to change organisational culture in health care, have been disappointing [87], and it has been argued that a focus on changing organizational climate may be more fruitful [81].

## The Role of Leadership and the Challenge of Diversity

### *Transformational leadership*

In general, leaders are viewed as having a strong impact on change processes in organizations [88] and are likely to play a critical role in effective implementation. Transformational leadership, one of the most heavily researched approaches to leadership, has particularly been tied to organizational innovation across a variety of studies, often through its influence on organizational climate [89–92]. Much in the same way that climate researchers have adopted a focused view of climates when predicting specific strategic outcomes, leadership researchers have begun to take a similar perspective [93–96]. Along these lines, researchers in the health services literature have recently developed a specific measure of implementation leadership [97]. This instrument appraises four dimensions of implementation leadership: knowledgeable leadership, supportive leadership, proactive leadership, and perseverant leadership. This research suggests that, it seems critical for leaders to have full knowledge of the innovation being implemented, to consistently show support for implementation efforts, to proactively plan for implementation efforts, and to persevere through setbacks and challenges in the implementation process.

### *Fostering inclusion and managing diversity*

The implementation of return-to-work programs for disabled employees also necessitates another important role for leaders beyond supporting implementation efforts: fostering inclusion of the disabled employees who are the target of the RTW program [98–100]. One mechanism through which leaders may enhance inclusion in their organizations is by creating a *climate of inclusion*, defined as “one in which policies, procedures, and actions of organizational agents are consistent with fair treatment of *all* social groups, with particular attention to groups that have had fewer opportunities historically and that are stigmatized in the societies in which they live” [101] (italics in original), which includes disabled employees [102]. A climate for inclusion includes fairness in implementation of employment practices, integration of differences, and inclusion in decision making [103], which promote employees’ needs for both belongingness and uniqueness being satisfied [101]. Although the importance of leaders in establishing inclusive workplaces has received some attention in the literature, evidence supporting this relationship is limited, Tetrick et al. [104] note there is evidence that consistency of leadership behaviour is important for establishing a climate of trust [105], but recommend further research specifically into the effectiveness of leadership as a component of organizational change effort and they cite organizational case studies to illustrate this in the context of attempts to enhance workplace wellness.

### Organizational Readiness and Organizational Change

Although leadership, in general, has been shown to be important for effective implementation [106, 107], efforts that do not consider both contextual and individual factors likely to facilitate or hinder implementation are likely to result in sub-optimal outcomes. In particular, Aarons et al. [108] argue that strategies that involve assessment, intervention, and support for implementation at multiple organizational levels should have a greater likelihood of success. *Organizational readiness* has been described in terms of its theoretical basis [109], its conceptualisation and measurement [110, 111] in terms of organizational members’ change commitment and change efficacy to implement organizational change [111], and in its utility in investigating influences on the implementation of worksite healthcare promotion programs [112]. More recent developments include a decision tool for assessing organizational readiness [113] and a detailed protocol for an organizational readiness intervention [114].

Franche et al. [25], however, have cautioned that in the area of work disability prevention there may be significant differences in the perception of roles and tasks between stakeholders (e.g. workers, management, health care providers). This suggests that such potential barriers should be identified and, if possible, addressed prior to implementation of the intervention [115]. Importantly, Franche et al. [25] also cautioned that perfect agreement between stakeholders may not be possible, and stakeholders may need to find ways to accommodate these differences.

### The Focus for Organizational Change

In addition to acknowledging the link between the organizational and individual perspectives [104], recognition of both the positive and negative aspects of the work environment may also be important [116, 117]. Recognition of the importance of the facilitation of positive adaptation to problems of ill-health and health-related work compromise has led to the view that well-being is not only an outcome of intervention, but also is a potential mediator of improved adjustment and performance. However, worksite wellness programs are characterized by complex pathways [112, 118, 119] and to date have shown only modest treatment effects [120]. Martins [121] has identified five major threats to the success of organizational change efforts: (a) lack of an adequate framework for implementing organizational change; (b) failure to accurately identify the problem; (c) inaccurate diagnosis of the problem and its root causes; (d) lack of fidelity in the implementation of a planned intervention; and (e) inadequate measurement of the resulting effect or insufficient time given. Although such challenges are not specific to worksite wellness programs, it would seem sensible at this time to defer further comment on their utility as an intervention for work disability until there is a clearer understanding of the mechanisms of change and in the implementation of interventions.

### Re-Engagement as a Component of Disability Management

Traditionally, disability management has been built on the three pillars of *prevention, work accommodation* and *support for recovery*, although how these have been implemented in different contexts has depended on policy at both a national/agency level and negotiated conditions of service and entitlements. There are also differences across jurisdictions in terms of legal responsibility of employers for sickness management in general and for specific injuries in particular [122]. In tackling work disability, there has been a major focus on *primary prevention,* (with worker centered education and instruction) and the minimization of danger, whether in the design of environment or in job design (in terms of its physical and psychological demands). Much of this can be developed of course at the level of the workplace and workforce. *Secondary prevention*, in terms of the interventions described in other chapters, may also be tackled at a workforce level, but at early stages typically will have involved clinical healthcare. In secondary prevention the primary focus has been on the amelioration of symptoms and re-integration into work, sometimes with phased return-to work or work modifications [19].

However, as mentioned above, there has been a change in understanding of the impact of work on health [2] and the introduction of wellness initiatives [104] has become a feature within many organizations. Implicit in many of the traditional approaches has been an assumption that “restoration” of physical and mental health will be sufficient to ensure return to work, but as mentioned earlier, interventions aimed at these targets have often failed when workplace factors have not also been addressed [22]. The process of work engagement/re-engagement may have multiple consequences for employees’ performance [123] and in organizational research there has been a broadening in perspective from *attendance management* to *sickness management* and a concomitant shift in emphasis from *symptom management* to the *enhancement of well*-*being*. It would seem that attention to facilitating engagement and re-engagement in work has the potential to assist those returning to work after illness or injury.

Engagement in work traditionally has not fallen within the purview of occupational health or the rehabilitation literature, but perhaps merits consideration in the context both of the enhancement of well-being and in pain management [18]. Schaufelli and Baker [124] acknowledge that the term “work engagement” has been understood somewhat differently in business and academia, but offer an integrative model linking characteristics of work (resourceful and challenging) and positive affect, with work engagement (characterized by job satisfaction and job involvement) with organizational commitment and with enhanced performance (evidenced, for example, in discretionary effort).

In summary, there is a case for reconceptualizing the challenge of work disability as one of sustained work re-engagement, often in the context of ongoing symptomatology, rather than one primarily of clinical cure or job redesign/accommodation. For such a shift in emphasis to gain any traction however, it is necessary to consider the challenge both from an employer’s and a worker’s perspective.

## Understanding the Employer’s Perspective

The majority of grey literature articles reviewed in the second paper in this special issue [26] did not address implementation issues per se, but they illustrated the kinds of arguments typically made to employers to support the uptake of more proactive disability management practices. These publications included summaries of best practices, case examples touting individual success stories, results of management benefit surveys, consultant advice to employers, and consensus-based guidelines. Examination of the documents with respect to employers’ rationales for implementation, suggested five recurring reasons for implementing more proactive disability management strategies, as described below:

### *Cost and productivity*

The most common appeal to employers was that more benevolent and proactive policies would show a positive return on investment by both reducing costs associated with sickness absence and by improving the overall productivity of the workforce. National estimates of disability-related costs to employers were frequently cited, and authors also made reference to the hidden costs in presenteeism, poor employee retention, and training of replacements. Several publications also made it clear that a business case would need to be made to senior members of the company for implementation of any new disability management policies or RTW programs. Overall, return on investment was likely to be the most important factor in organizational decision-making around disability issues.

### *Legal compliance*

The second most frequently cited reason for adopting more proactive disability management strategies was to remain compliant with changing laws and regulations and to avoid lawsuits and accusations of discrimination. In addition to the high costs of fines and legal action, authors mentioned the negative effect of recurrent legal action on workplace morale and labor-management relations that could lead to additional losses in productivity and turnover.

### *Competitive advantage*

The third most frequently cited reason was to emulate model employers and to keep up with the practices of successful competitors. Adopting more proactive disability management policies might help to portray an organization that is up-to-date, progressive, and innovative. In addition to attracting new employees, the authors also cited its potential impact on consumers and investors and a more positive public image of the company.

### *Employee well*-*being*

:Fourth, there was frequent mention of the positive impact of disability management programs on worker well-being. For injured or ill workers, these benefits were described in terms of both material outcomes (e.g. less time on partial wage replacement, less risk of unemployment) and in terms of personal well-being (e.g. feeling more functional, less impaired, less stigmatized). For the workforce in general, proactive disability management policies might help to foster a culture of inclusivity and fairness. In several cases, this was described as a “win–win” proposition that benefitted both employers (by reducing costs) and employees (by improved well-being).

### *Administrative efficiency*

Finally, another point in support of better disability management practices was to address administrative gaps and a possible lack of uniformity in the treatment of disability problems. Smoothing of administrative wrinkles appeared to be a viable case for improved disability management practices simply from the perspective of fairness and efficiency. Some employers, then, may be willing to implement new disability management strategies from a concern that absences and accommodations are not being properly or fairly tracked and supervised.

These five reasons expressed in the recent grey literature for employers to implement proactive disability management practices closely match those formulated by Akabas and colleagues [125] who list seven arguments that could be employed to support the case for more proactive disability management practices: (a) improve competitiveness of the company; (b) achieve a healthier and more productive workforce; (c) reduce medical and disability costs; (d) shorten or reduce the disruption of sickness absence; (e) reduce the personal burden to employees; (f) enhance morale by valuing diversity; and (g) achieve regulatory compliance.

In addition to identifying organizational facilitators, some of the grey literature articles also noted potential organizational barriers. For example, Batterson et al. [126] listed several common frustrations expressed by employers about the implementation of modified duty programs: (a) “We do not have any modified-duty jobs”; (b) What if an employee’s condition gets worse by coming back early?”; (c) “I have a lot of work to be done. I need everyone to be able-bodied”; (d) “The budget does not allow for ‘extra’ employees”; (e) “Modified duty is bad for morale or encourages favouritism”, (f) “The program is too time-consuming to administer”; (g) I cannot have everyone permanent light-duty assignment”; (h) “The program costs too much”; and (i) “The union will never agree to this”. Clearly, such employer concerns about cost, fairness, morale, and job modification need to be heeded and addressed. Future research may assist in providing evidence for counter-arguments to these reservations.

A final point that may be drawn from the grey literature is the extent to which employers perceived they were often excluded from the disability benefit system and seemed to be effectively placed outside of the policy process as well, with most of the focus instead on healthcare providers and social insurance systems. Despite the evidence provided by Franche et al.’s [25] review of the importance of linkages between employers and healthcare providers, employers seem to still be viewed as part of the problem, not part of the solution. This is likely to result in missed opportunities for early employer-led disability prevention efforts during periods of initial time away from work. One publication [127] provided an excellent statement of this problem: “Not only employers, but also administrations, workers’ representatives and doctors, seem to lack sufficient knowledge about such workers in order to prevent them from gradually sliding into sickness and, later, disability benefits.” (pp. 14). Changes were proposed to the roles and incentives to employers, the supports and tools available to employers, and the need for better communication with other stakeholders.

This was also illustrated in a study from the Burton-Blatt Institute which contrasted employers reporting formal versus informal return-to-work programs [128]. An internet-based survey was completed by managers from 232 companies. The survey included a variety of organizational factors that were then compared between employers with formal (45 %) and informal (55 %) RTW programs. For those employers with formal RTW programs, their principal reasons for adopting this approach were: (1) to reduce lost time costs; (2) based on moral obligation (“the right thing to do”); and (3) protecting their investment in their workforce. For employers with more informal RTW programs, their reasoning was that this represented a simpler and more flexible approach. Perceived strengths of existing programs concerned issues of communication, professional knowledge, and consistency/fairness. Areas reported to be in need of further development were physician and supervisor communication, increasing accessibility to workers with disabilities, and expanding the breadth of the program. Somewhat surprisingly, 42 % of respondents had no systematic method in place for evaluating their programs, and 78 % had no way of measuring return-on-investment. Despite this, when asked what was necessary to encourage more proactive practices, respondents indicated (1) evidence of return on investment, (2) a need to meet stricter regulatory requirements, (3) an internal champion; and (4) an adjustment in senior management priorities. These results suggest that the organizational appetite for disability management practices has as much to do with managerial priorities and corporate culture as with bottom-line financial issues.

## From Abstraction to Reality: a Case Study Using the CFIR

The Aarons et al. [47] model can also be illustrated by use of a case example. Our case example is the implementation of an early risk screening and psychosocial management intervention instituted for employees with acute soft tissue injuries within a large hospital network in Australia. The screening used the 10-item Orebro Musculoskeletal Pain Screening Questionnaire (OMPSQ-10; [129]) which was administered over the phone by the insurance case manager within the first week of an injured worker taking time off work due to their musculoskeletal injury. Those scoring above the cut-off of ≥50/100 were offered the opportunity to address their concerns with a nominated psychologist (in addition to usual care by their treating doctor and physical therapist). Any work-related issues identified by the psychologist or workplace return to work coordinator were to be addressed simultaneously at the workplace. The protocol incorporated collaborative input from the key stakeholders (workplace, insurer, treatment providers, and injured worker). The main features of the study are summarised in Fig. 3. While this study has only just been completed, the employer (the NSW State Health Department) has recognized its value (to date the savings have amounted to 22 % for the high-risk intervention group over the similar control group, and mean lost work days of 30 vs 56, respectively, over the year following injury) and it is now being implemented as standard practice for all public hospitals in that state. In addition, planning is underway to change the guidelines covering the early management of injured workers generally across the state. The project provides a practical example of how the multi-level Aarons et al. model [38] can be used to address likely implementation barriers within a complex workers compensation insurance environment. Final results of the study will be available in early 2017.

**Fig. 3:**
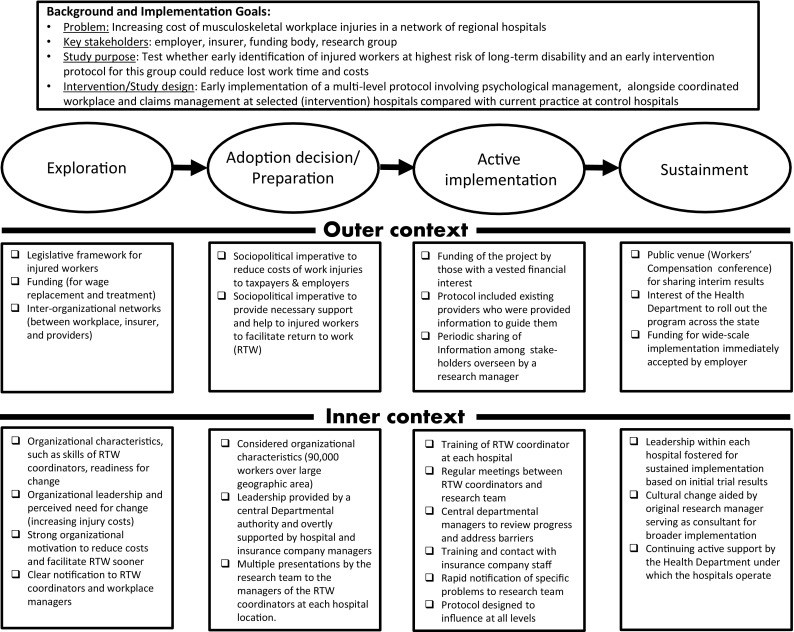
Case study of an on-going screening and early pain management program being implemented in a network of regional hospitals

## Conclusions

This paper has drawn on innovations from Implementation Science to address the question of how a more effective and sustainable RTW outcomes for injured workers might be achieved. Evidence from a search of the occupational rehabilitation literature, employer challenges described in the grey literature, and a recent study case example indicate that the framework by Aarons et al. [45] has some applicability to work disability prevention strategies in the workplace. We conclude that there are two overarching issues of particular importance in the design and implementation of interventions in the workplace: *implementation strategy* and the *context of implementation*.

### *Implementation strategy*

Aclear message from this review is that successful implementations in the workplace need to be planned, with clear specification of the desired outcomes and inclusion of a strategy for a coordinated, multi-level intervention. As indicated earlier in this paper, there are many ways in which this might be undertaken, but as a starting point, some recommendations are offered in Table 1.

It has been suggested that the undertaking of specific tasks can be aided by the use of a comprehensive model, such as the one described by Aarons et al. [47]. The choice of intervention, of course, depends on the nature of the presenting problem and as well as the desired outcome, which in many business organizations is frequently gauged in terms of ROI (Return-on-Investment). Here we have attempted to outline the issues which need to be considered in optimizing the implementation of interventions and their sustainability.

### *Implementation context*

Using the Aarons’ et al. model, it has been argued that a specific RTW intervention for an injured worker should be seen as but one element, set within both an Inner Context (comprising multiple levels, all with different relationships and interactions between them, from the individual worker’s immediate co-workers to their supervisor, the supervisor’s manager and ultimately the Managing Director or CEO of the company/organization) an Outer Context (which may include multiple providers and their relationships and interactions, as well as the legislative framework covering workplace injuries, and even a given society’s expectations) and connections between them (such as the role of insurance carriers, the funding of the provision of rehabilitation and other such contextual factors). It has been suggested further that a distinct and discrete focus on the *phase* of implementation will enable a still clearer focus on the implementation. It has also been acknowledged that while models like that of Aarons et al. [45] may provide a helpful framework for categorizing the sorts of issues that may be important to consider when planning to implement RTW research within the workplace, they do not provide guidance on *how* these steps might be undertaken. For this we turned to evidence from occupational rehabilitation, organizational psychology and well-being research.

In designing interventions for work disability in the workplace, the literature would suggest that three initial considerations appear to be of particular relevance: first, the prevailing organizational culture and climate; second, the nature of the leadership style in the organization (which may require several dimensions, from being supportive to inclusive, to assisting with persistence, across the period of implementation); and third, the degree to which the organization in question seems ready for change. Each of these features or characteristics are likely to require different approaches. Underpinning many of these features is the importance of identifying the employer’s perspective, and this can be reflected in areas like their view of re-engagement of injured or disabled workers, as well as the more traditional issues like costs, productivity, legal compliance, administrative efficiency, and management priorities. Recent research into the nature of knowledge translation identifies it as an important element in RTW interventions and of course this can be influenced by factors such as who is providing the information and the perception of that person held by the recipients of the knowledge translation.

In conclusion, it has been suggested that in the light of problems in workplace implementation of RTW research a new perspective is needed for the design and implementation of research into workplace interventions for work disability. It has been recommended that a shift in perspective from a specific worker-centered focus to a broader contextual view of work disability, with consideration specifically of the determinants of work re-engagement, may offer the opportunity to develop more effective interventions, build in the engagement of all key stakeholders thus enhancing the effectiveness of the implementation and producing change which is likely to be sustained.

## Footnotes

1 The concept of presenteeism [51] is well known in the occupational literature and has been the subject of a recent review [52]. Although measured in a number of ways and defined at time with differing emphases, it can simply be understood as “Working when ill” and has been associated with sub-optimal performance

2 Similarly, “absence management” is understood by us to refer to the organizational systems and practices associated with workloss (in this context primarily associated with work compromise secondary to injury or illness).
